# The Design Matters: How to Detect Neural Correlates of Baby Body Odors

**DOI:** 10.3389/fneur.2018.01182

**Published:** 2019-01-16

**Authors:** Laura Schäfer, Thomas Hummel, Ilona Croy

**Affiliations:** ^1^Department of Psychotherapy and Psychosomatic Medicine, Technische Universität Dresden, Dresden, Germany; ^2^Smell and Taste Clinic, Department of Otorhinolaryngology, Technische Universität Dresden, Dresden, Germany

**Keywords:** fMRI design, olfaction, olfactory fMRI, body odor, baby odor, body odor perception

## Abstract

Functional magnetic resonance imaging of body odors is challenging due to methodological obstacles of odor presentation in the scanner and low intensity of body odors. Hence, few imaging studies investigated neural responses to body odors. Those differ in design characteristics and have shown varying results. Evidence on central processing of baby body odors has been scarce but might be important in order to detect neural correlates of bonding in mothers. A suitable paradigm for investigating perception of baby body odors has still to be established. We compared neural responses to baby body odors in a new to a conventional block design in a sample of ten normosmic mothers. For the new *short* design, 6 s of continuous odor presentation were followed by 19 s baseline and 13 repetitions were performed. For the conventional *long* design, 15 s of pulsed odor presentation were followed by 30 s of baseline and eight repetitions were performed. Neural responses were observed in brain structures related to basal and higher-order olfactory processing, such as insula, orbitofrontal cortex, and amygdala. Neural responses following the short design were significantly higher in comparison to the long design. This effect was based on higher number of repetitions but affected olfactory areas differently. The BOLD signal in the primary olfactory structures was enhanced by short and continuous stimulation, secondary structures did profit from longer stimulations with many repetitions. The short design is recommended as a suitable paradigm in order to detect neuronal correlates of baby body odors.

## Introduction

Neural processing of social stimuli has been well studied for the senses of vision and audition, but examination of interpersonal human chemosensation is just in the beginning due to challenges related to the olfactory system.

The detection of reliable neural activations to odors is complicated due to the anatomical structures of the olfactory system and methodological obstacles related to the presentation of olfactory stimuli (1). We briefly outline those challenges.

Central olfactory processing occurs in several stages [compare 1]. Olfactory signals coming from the olfactory bulb (OB) pass on to the basal frontal and medial temporal lobe. Thereby, the piriform cortex, the amygdala, the perirhinal and entorhinal cortices receive parts of the incoming information from the OB (2). Those areas are commonly considered as primary olfactory areas (3). From there, olfactory information is further processed in secondary structures, such as the anterior insula, hippocampus, hypothalamus, and orbitofrontal cortex (OFC). In contrast to other modalities, olfactory processing is characterized by direct pathways projecting into primary and secondary structures without passing through the thalamus first. Due to the subcortical structures involved in olfactory processing, the detection of olfactory signals in functional magnetic resonance imaging (fMRI) is challenging. The olfactory system is surrounded by the frontal and the paranasal sinus, and the acoustic meatus containing various tissues (bones, vessels, air) with different magnetic field homogeneity characteristics (1). Those make this system especially sensitive to susceptibility artifacts and limit signal detection in the mediobasal parts of the brain. Although well-adjusted fMRI sequences can reduce those artifacts, a systematic overview of the most suitable procedures is still missing.

Another difficulty in olfactory fMRI is the odor presentation: stimulus concentration and duration are typically operated by computer-controlled olfactometers, which are stationed outside the scanner and deliver odors via several meters of tubing to the participants' nose. Thus, presenting precise stimulus onsets is challenging. Particular devices, e.g., portable olfactometers, facilitate stimulus presentation, as they allow the odors to be placed close to the MRI scanner or within the scanning room [e.g., 4].

Besides that, the rapid adaptation to olfactory stimuli needs to be considered (1) and the length of the olfactory stimulation period as neural oscillations occurring after a longer stimulation time may affect the signal (5).

In addition to those general challenges of olfactory fMRI, the stimulation with *body* odors has particular demands: Body odors are generally weak and not easily producible or storable in high concentration as compared to other, e.g., liquid odorants. Typically, clothes worn by the subject serve as body odor stimuli, but the amount of odor molecules within such a piece of clothing is limited. This weaker concentration of molecules may explain the weaker neural activation compared to other olfactory stimuli.

Further, the field of studies investigating neuronal processing of body odors is small and lacks conventions about optimal designs. To our knowledge, only four original fMRI investigations on body odor perception exist (compare Table 1). Two used a block design with about 20 s of pulsed odor presentation (6, 7); the other two used an event-related design with about 3 s of continuous odor presentation (8, 9). All four studies report weak activations in general and in some studies the expected olfactory areas were not observed at all. Further studies based on positron emission tomography [PET, 10, 11], or near infrared spectroscopy [NIRS; 12] report similar, and again, weak effects (see Table 1).

**Table 1:** Comparison of olfactory imaging studies investigating neural correlates of body odor perception.

	**Experimental parameters**							**Odor presentation**			**Activation due to odor (vs. baseline)**	
**Study**	***N***	**Design**	**fMRI parameters**	**Number of conditions**	**Repetitions per condition**	**Duration (s); ISI (s)**	**Mode of presentation**	**BO sampling**	**Breathing**	**Airflow**	**Range of** ***T*****-/*****Z*****-values**	**Results**
Lübke et al. (6)	14 women: high social openness; 12 women: low social openness	fMRI: block design	1.5 T; TR: 2.5 s, TE:40 m; FOV: 192 × 192 mm; matrix: 64 × 64	4 (each 2x male and female BO)	6	22; 22	Pulsed (2 s on, 2 s off)	Axillary sweat	Velopharyngeal closure technique	2l/min	T: 3.71–5.43	BO > BL: SVC: fusiform cortex, ACC, PCC, insula
Lundström et al. (7)	15 women: postpartal;15 women: nulliparous	fMRI: block design	1.5T; TR: 2.63 ms, TE:45 m, FOV: 24 slices axial plane oriented parallel to the planum sphenoidale; matrix: 64 × 64	2 (2x different BO infant)	12	20; 20	Pulsed (1 s on, 4 s off)	Cotton shirts (infants)	Velopharyngeal closure technique	3l/min	Z: 3.64–4.53	BO> BL: hippocampus, insula; SVC: lateral orbitofrontal cortex, putamen, ventral caudate nucleus, dorsal caudate nucleus
McGlone et al. (8)	21 women	fMRI: event-related design	3T; TR:1.5 s, TE:30 ms FOV: 192 × 192 mm; matrix: 64 × 64	2 (pleasant male fragrance; unpleasant artificial BO)	10	2.5; 16.5	Continuous	Artificial BO: thiol compound; male fragrance	Cued breathing (stimulus onset)	4l/min	Z:2.13–2.63	Fragrance < BO: OFC; BO > fragrance: piriform cortex, amygda, frontal operculum, supramarginal gyrus, thalamus
Prehn-Kristensen et al. (9)	28 students: 14 women	fMRI: event-related design	3T; TR:3.25, TE:35 m, FOV: 40 transversal slices covering the whole brain; matrix: 80 × 80	4 (BO anxiety, BO sport; each 2x male and female)	20	3; 17.8	Continuous	Axillary sweat: anxiety and sport condition	Online visual inspection of breathing belt	3l/min	Z: 3.63–5.13	Smelled BO > non-smelled BO: post-central gyrus, medial temporal gyrus, thalamus, putamen, dorsolateral frontal gyrus
Lundström et al. (10)	15 women	PET	–	5 (no odor BL, odor control, BO self, BO friend, BO stranger)	20	3; 5	Continuous	Axillary sweat	Cued breathing (stimulus onset)	–	T: 2.7–4.24	BO > BL and BO > odor control: PCC occipital gyrus, angular gyrus; dorsal medial ACC, angular gyrus
Lundström et al. (11)	12 women: nulliparous	PET	–	4 (no odor BL, odor control, BO sister, BO friend)	20	3; 5	Continuous	Axillary sweat	Cued breathing (stimulus onset)	–	T: 3.5–5	BO > BL: medial cingulate cortex, ACC, superior frontal gyrus, posterior medial frontal gyrus, dorsomedial prefrontal cortex, fronto-temp junction, postcentral gyrus, PCC, angular gyrus, occipital cortex, parietal operculum, insula, culmen
Nishitani et al. (12)	19 women: postpartal; 19 women: nulliparous	NIRS	–	6 (3x different BO infant, 3x unworn cotton shirt)	18	5; 5	Continuous	Cotton shirts (infants)	Cued breathing (stimulus onset)	–	–	BO > BL: PFC activity

*fMRI, functional magnet resonance imaging; PET, Positron emission tomography; NIRS, Near infrared spectroscopy; BO, Body odor; FOV, Field of view; BL, baseline; ISI, inter-stimulus interval; s, seconds; min, minute; SVC, small volume corrected; ACC, anterior cingulate cortex; PCC, posterior cingulate cortex; OFC, orbitofrontal cortex; PFC, prefrontal cortex*.

Besides olfactory areas, both the anterior and the posterior cingulate cortex (ACC, PCC) have been associated with body odor perception (6, 10) and it was supposed that the processing of endogenous (body-) odors differs from exogenous odors and activates other brain areas apart from the olfactory system (10).

To our knowledge, only two imaging studies have investigated *baby* body odor perception in mothers [fMRI: 7; NIRS: 12]. Baby body odors are subtle which implicate that investigations and the detection of strong neural effects are especially challenging. The present study was conducted in order to investigate which design characteristics are particularly suitable for imaging neural responses to baby body odors.

We designed a new, short block presentation paradigm aimed to account for rapid adaptation (by shortening odor presentation time to 6 s) and for weak neural responses following body odors (by increasing the number of stimulus repetitions). We compared this to a long block design, which follows recent recommendations (1) with 15 s of odor presentation; hereby the odor presentation was performed in a pulsed way to overcome adaptation. Our targeted outcome was the strength of neural activation in olfactory relevant brain areas depending on the design. According to previous results, we focused our analysis on the anterior insula, the OFC, the piriform cortex and the thalamus, as well as on the ACC and PCC. We furthermore included the amygdala and the hippocampus as regions of interest (ROI) which are frequently activated in response to odor presentation.

## Materials and Methods

The ethics committee of the University of Dresden (Code: EK 104032015) approved the conduction of the study according to the “World Medical Association's Declaration of Helsinki.” Written, informed consent was obtained from all participants.

### Participants

Our sample consisted of 10 healthy, normosmic mothers (aged 27 to 39 years, *M* = 32.2; *SD* = 4.7) having a child under the age of 2 years (aged 10 to 15 months, *M* = 10.30, *SD* = 4.22). Normosmic functioning was ensured with a Sniffin' Sticks identification screening (13). This study was done as a pilot measurement for a larger project.

### Magnetic Resonance Imaging Procedures

Functional magnetic resonance data were acquired on a Siemens 3T scanner SONATA with an 8-channel head coil using a protocol with a T2^*^-weighted gradient-echo, echo-planar imaging sequence (*TR* = 2.5 s, TE 51 ms, flip angle 90°, 25 mm × 6 mm axial slices, 3.6 × 3.6 mm in-plane resolution). In order to receive a precise anatomical mapping of the functional data, a high resolution T1 sequence (TR = 2.5 s, 0.7 × 1mm in-plane resolution) was added. The scanning planes were oriented parallel to the anterior-posterior commissure line and covered olfactory relevant regions from the cerebellum up to the dorsal end of the cingulate cortex. As all areas dorsal to the cingulate cortex were no regions of interest in the present study, we decided to limit the scanned area of axial sections from the brain stem up to the cingulate cortex (*z* = 45 at *y* = −80 to *z* = 20 at *y* = 60) in order to enhance the repetition time and to allow for more scans during the session.

### Body Odor Sampling and Presentation Procedure

Body odor samples were collected with onesies worn for one night by the babies after a standardized procedure (see Supplementary Material). The armpit of the onesie was stored in a glass bottle connected with teflon tubes (5 m length) to the air-dilution computer controlled olfactometer (4).

Two different designs of odor presentation were used. Both lasted for the same time of 6 min, but differed in the duration, mode, and number of repetitions of odor presentation within (Supplementary Figure 1). This was done in order to match previous design characteristics which used either block designs with long pulsed stimulus presentation (6, 7) or event-related designs with short continuous presentation (8, 9).

Hence, we used a long pulsed block design and compared this to a short odor presentation. The short was similar to previous event-related designs in terms of a short continuous presentation but differed as we did not jitter and randomize the olfactory stimuli within the run. We refrained from that in order to not over complicate the comparison with additional variables as study power was limited.

In the long design, 8 on-blocks of 15-s each in which the odor was delivered were followed each by 8 off-blocks of 30-s each. Due to the long on-blocks, a pulsed odor presentation, where 2 s of air followed every 1 s of odor presentation, was used in order to minimize adaptation and habituation to the odors. In the short design, 13 on-blocks in which the odor was continuously delivered for 6 s were followed each by 13 off-blocks of 19 s each. Each paradigm was tested with two different stimuli in randomized order: the body odor of the own baby and an unfamiliar sex- and age-matched child, resulting in four runs in total. During baseline, clean air was presented. As the main focus of the present study was to compare the design paradigms, the effect of baby body odor was merged across own and unfamiliar baby for statistical analysis. Single results of own and unfamiliar child are provided in Supplementary Tables 3, 4. Before the experiment, participants were instructed to breathe regularly through the nose as follows: “You are presented to baby body odors, one of which is your child. Please, breathe regularly and smoothly as normally through the nose.” After each run, participants rated pleasantness, intensity, and wanting of the odor stimuli on a Likert-scale ranging from 1 = “not pleasant/intense/not at all” to 10 “very pleasant/intense/very much.” Pleasantness and wanting reflect different characteristics of reward (14). Wanting thereby indicates the incentive value of the stimulus and was assessed with the item asking “How much would you like to smell the odor again?,” whereas pleasantness displays the hedonic aspect and was assessed by the question “How pleasant is this odor?” In addition, the mothers were asked if the presented odor belonged to their own child (“yes/no/I don't know).” Answers of the behavioral ratings are provided in Table 2.

**Table 2:** Mean values and standard deviations for ratings of pleasantness, intensity, and wanting for each design for the single (own, unfamiliar) and merged across own and unfamiliar baby body odor, *T*-Test comparison of the merged (across own and unfamiliar) baby body odor ratings between short and long block design (*t*, df, *p*-value).

	**Short block design**						**Long block design**						***t***-**test: merged ratings (short vs. long block design)**		
**Baby odor**	**Own**		**Unfamiliar**		**Merged**		**Own**		**Unfamiliar**		**Merged**	
**Parameters**	***M***	***SD***	***M***	***SD***	***M***	***SD***	***M***	***SD***	***M***	***SD***	***M***	***SD***	***t***	***df***	***p***
Pleasantness	7.30	1.77	6.6	2.01	7.12	1.79	5.70	2.90	6.33	2.34	6.00	2.60	1.97	18	0.065
Intensity	3.70	2.21	4.70	2.91	4.37	2.52	3.40	2.37	4.33	2.87	3.84	2.59	1.06	18	0.304
Wanting	5.70	2.79	5.40	2.91	5.84	2.52	4.20	3.01	6.44	2.12	5.26	2.81	1.07	18	0.300

*N = 10; the variables are rated on a 10-point Likert scale; with lower values indicating less and higher values indicating higher pleasantness, intensity, and wanting. When the own baby body odor was presented, two mothers after the short block and one mother after the long block run stated to recognize their own child. When the unfamiliar baby body odor was presented, one mother after the short block and none of the mothers after the long block run stated to recognize their own child. In addition to the comparison reported, we also tested the following contrasts using t-tests for each rating: own (short) vs. unfamiliar (short) and own (long) vs. other (long). No significant differences between the baby odor stimuli were observed*.

### Data Analysis

Data was analyzed with SPM 12 (Wellcome Trust Center for Neuroimaging, London, UK, implemented in Matlab R2014b; MathWorks, Inc., Natick, MA, USA). The preprocessing was done identically for both designs with the default settings used in SPM 12 and comprised realignment with 2nd degree B-spline, unwarping with 4th degree B-spline, and co-registration by segmentation fitting to the individual T1 volume. The images used for analyses were spatially normalized (stereotactically transformed into MNI ICBM 152-space) and smoothed with a Gaussian kernel of 6 mm FWHM.

For the first level analyses, we started with the two sessions performed with the short design: The full 13 stimulation periods were contrasted to the full (13 × 6 s = 78 s) subsequent off-period (13 × 19 s = 247 s, compare Supplementary Figure 1). We named this contrast “short_full_.”

For both sessions performed with the long design, the whole on-period (8 × 15 s = 120 s) was contrasted to the whole subsequent off-period (8 × 30 s = 240 s). We named this contrast “long_full_.”

As the short design comprised more repetitions than the long design, we performed an additional analysis. In order to match the number of repetitions between both designs, we analyzed only the first 8 on- and off–blocks from both sessions. We named this contrast “short_reduced_.”

**Figure 1:**
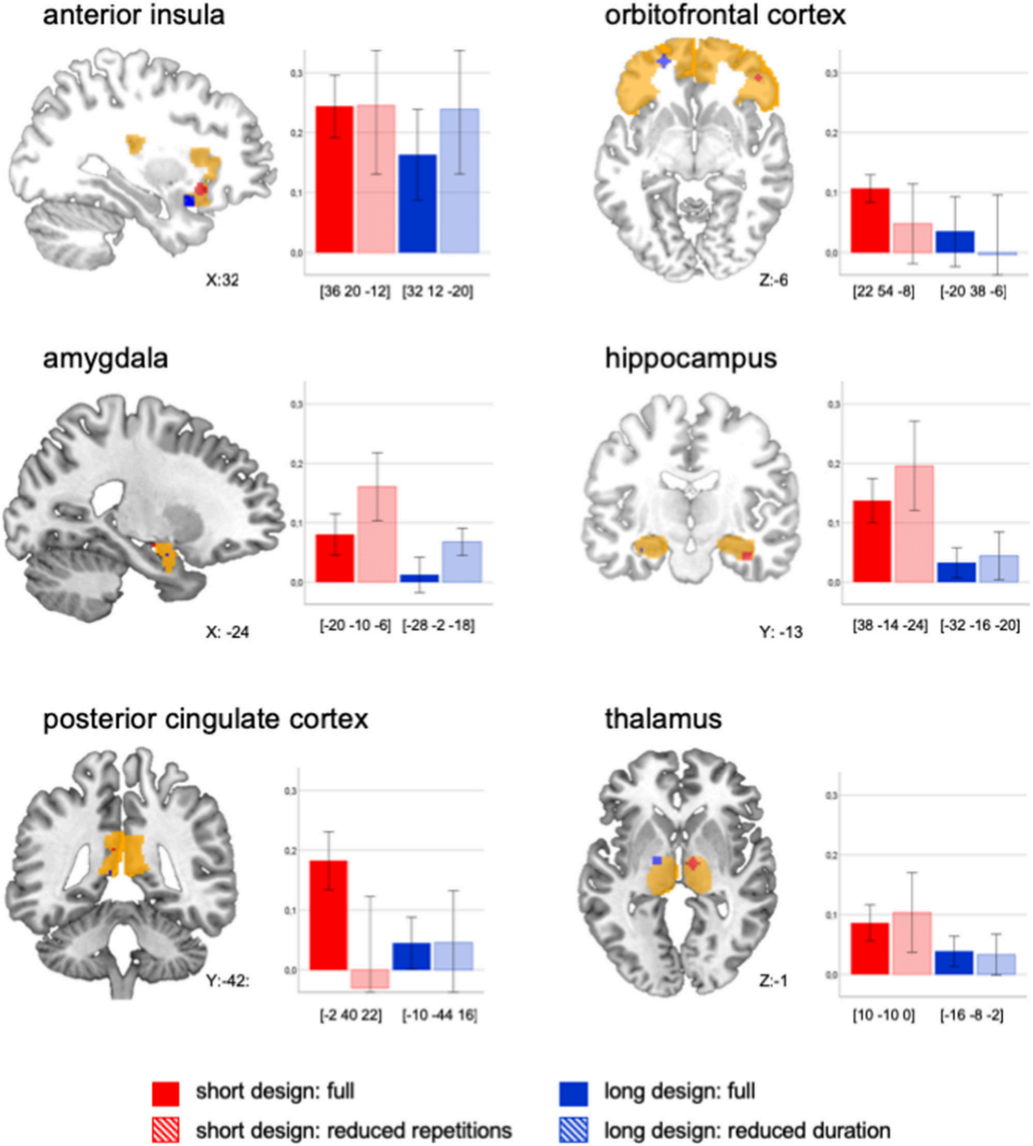
Peak activations displayed for each ROI and each design. Beta mean values (baby body odor vs. baseline) extracted for a 4 mm sphere around the peak value (RH, right hemisphere; LH, left hemisphere, and MNI coordinates are displayed in square brackets) of each ROI and for each design across all subjects (*n* = 10). Please note in the anatomical visualization, that peak activations may have occurred on different hemispheres. Error bars display 95% CI.

As the long design was characterized by longer stimulus delivery than the short design, an additional analysis was performed. In order to match the stimulation duration, only the first half of the on-period was used (8 × 7.5 s = 60 s) and compared to the whole subsequent off-period (compare Figure 1). We named this contrast “long_reduced_.”

For the second level analyses, four t-contrasts with one-sample *t*-tests were computed for the overall effect of the baby odor (on-period merged across own and unfamiliar baby vs. off period, clean air) for each design and analysis approach (short_full_, long_full_, short_reduced_, long_reduced_) in order to detect general activations related to the odor presentation across all subjects.

As the main aim of this study was not the determination of neural activations, but the exploration of the best suitable design characteristics, the comparison between both designs was based on the signal strength within a given ROI. ROI analyses were performed for the following regions: Anterior insula, OFC, amygdala, hippocampus, ACC, PCC, piriform cortex, and thalamus. ROIs were built with WFU Pick Atlas 3.0.3 (15) toolbox for SPM (for details, see Supplementary Material). ROI analyses were performed contrasting the effect of the baby body odor to the baseline condition. For each ROI in each design (apart from the ACC and the piriform cortex where no supra threshold activations were observed), the mean beta signal across all subjects was extracted for a 4 mm sphere around the peak voxel using MarsBar (16).

Subsequently, a generalized linear mixed model (GLM) was performed (IBM SPSS Statistics 25) in order to test the effect of the design on the signal strength. Each participant (*n* = 10) served as an individual, each stimulus (own and other baby) and each ROI (anterior insula, OFC, amygdala, hippocampus, PCC, thalamus) served as repeated measurement. The extracted mean beta signal was used as target for the main effect of the design across all ROIs.

We contrasted the new to the conventional design (short_full_ vs. long_full_). Afterwards, we systematically compared the different analysis approaches to each other in order to specify whether this effect was based on the number of repetitions, duration (length of stimulation period) or mode (continuous or pulsed stimulation) of the presentation. For effect sizes, we calculated Cohen's d. Results within the ROIs are descriptively reported.

In order to explore additional activations following baby odor stimulation, a whole-brain analysis was performed for the strongest design (short_full_). The effect of baby body odor (merged across own and unfamiliar baby) was contrasted to the baseline with a threshold of *p* < 0.001 (uncorrected) and a cluster extent threshold of *k* > 20 (Supplementary Table 2, Supplementary Figure 1). Analyses of the single effects of each baby body odor (own baby vs. baseline; unfamiliar baby vs. baseline) are presented in the Supplementary Material (compare Supplementary Tables 3, 4, Supplementary Figure 2).

## Results

### ROI Analyses

There were superior BOLD signal activations in the short design compared to the long design across all ROIs [short_full_ vs. long_full_: *F*_(1, 22)_ = 8.67, *p* = *0.0*07, *d* = *0.3*4, see Figure 1]. We aimed to systematically compare whether this effect was based on number of repetitions, duration, or mode of presentation.

The comparison between the long_full_ to the short_reduced_ design indicated an effect of the number of repetitions: When both designs had the same number of repetitions, the short was not superior to the long design anymore [*F*_(1, 13)_ = 1.74, *p* = 0.220). The comparison of the short_full_ to the long_reduced_ design indicated no effect of stimulation duration: When both designs had the same duration, the short was still superior to the long design [short_full_ vs. long_reduced_: *F*_(1, 159)_ = 15.61, *p* < 0.001, *d* = 0.24).

Thus, the observed superiority of the short design could be either due to *number of repetitions* or to the *mode* of presentation. In order to explore this further, we statistically compared the designs changing the parameter of interest (number of repetitions, mode, duration) and keeping the other two elements constant:

The direct comparison of number of repetitions, when keeping duration and mode constant, did not show a significant effect across the ROIs [short_full_ vs. short_reduced_: *F*_(1, 18)_ = 0.01, *p* = 0.922]. Visual inspection revealed a differential effect: A high number of repetitions led to lower BOLD signal in amygdala and hippocampus, but to higher signal in secondary structures, namely the OFC and PCC (Figure 1).

The direct comparison of *mode* when keeping number of repetitions and duration constant, did not show a significant effect across all ROIs [short_reduced_ vs. long_reduced_: *F*_(1, 21)_ = 1.59, *p* = 0.221]. Visual inspections showed a more differential effect, so that continuous presentation led to a higher signal in all ROIs except for the PCC and the anterior insula (Figure 1).

The direct comparison of *duration* when keeping number of repetitions and mode constant, did not show a significant effect across the ROIs [long_full_ vs. long_reduced_: *F*_(1, 41)_ = 0.67, *p* = 0.419]. Visual inspection showed—again—a more differential effect: a reduced duration of stimulation led to higher signal in amygdala, hippocampus and anterior insula, but to lower signal in the OFC (Figure 1).

### Whole Brain Analyses

Whole brain analysis was performed for the paradigm with the strongest neural activation (short_full_) and revealed rather weak responses in a total of four significantly activated areas, namely the superior temporal gyrus (STG), the OFC, the brain stem, and the anterior insula (compare Supplementary Table 1).

## Discussion

Our results demonstrated superior activations in the short compared to the long design across ROIs. Systematic analyses revealed differential effects on olfactory areas depending on number of repetitions, duration, and mode of the stimulation. The clearest results were observed for the amygdala: for this structure, considered as part of the primary olfactory cortex (3), it seems beneficial to design body odor stimulation with fewer repetitions per run, shorter duration, and continuous presentation. We assume that this effect is due to the rapid habituation and adaptation in primary olfactory areas (17). To overcome the early habituation and preserve power, we suggest a higher number of short runs. Alternatively, stimulation with long and jittered inter-stimulus intervals can be recommended, though this will increase the total duration of the design.

For subsequent and later habituating structures, namely the OFC, many repetitions and long stimulation seem to be beneficial. Such an approach was implemented in the long design. However, great care has to be exerted in order to achieve a sufficient number of repetitions with this design. An optimal combination of long stimulation and high number of repetitions should be weighed. Based on our data we suggest 15 s of stimulation and at least 13 repetitions.

Taken together, our study showed diverse effects on different brain areas. A reduced stimulation duration for instance led to stronger signal in amygdala, hippocampus, and anterior insula, but to weaker signal in the OFC. This matches previous research showing that BOLD signal of hippocampus and anterior insula have similar time courses, while the BOLD signal time course of the OFC is delayed (17). The authors attributed this to the high interconnections, which result in similar patterns between the former structures. The OFC receives likewise direct input from primary olfactory areas (3). Additional incoming information via the thalamic pathway may explain its prolonged response (17). Hence, particular design characteristics should be considered with regard to the areas of interest.

A recent study (5) suggested a benefit of a high number of repetitions and short stimulation duration due to oscillations in the neural signal, which only occur after longer duration. Our study partly supports this assumption, as the combination of short and continuous stimulation with higher number of repetitions showed strongest activations. Yet, this effect could not be linked to the short duration, but rather to differential effects on primary or secondary structures depending on the respective combination of design characteristics.

The comparison in our study refers only to a *block* design. The short design was in fact similar to an event-related design in terms of short continuous stimulation alternating with rather long off-periods (8, 9). However, the stimuli were not randomized within a run; the stimulation was longer than in conventional event-related designs and on-off-periods alternated in the same interval. Further research comparing the short with a randomized and jittered design might be informative.

We are aware that the explanatory power of the study is limited due to the small sample size. However, we like to briefly review the additional results. Beyond the olfactory regions, presentation of baby body odors activated the PCC, as well as the STG. The PCC has been related to social chemosignaling (10), which matches our findings. As the STG is important for social cognition (18), the observed activation in our study might be referred to the social relevance of the baby odor stimuli.

The smell of the own baby is crucial for mother-child interactions and facilitates kin recognition and bonding in many species. In humans, higher reward-associated neural responses to baby body odors were observed in mothers compared to non-mothers (7) and it was suggested that maternal bonding is moderated by olfactory cues. The present study aimed to work out a suitable design for the detection of neural correlates to baby body odors. It provides the ground to examine the differences of neural processing of body odors from the own vs. other children.

## Conclusion

There is no common paradigm for the detection of neural correlates to body odor perception and the few studies performed in this area showed diverse results. The present study was conducted in order to find optimized design paradigms for presenting baby body odors in the fMRI and results may transfer to general body odor perception. As the short design revealed superior activations, we recommend this as a time-efficient and effective paradigm.

## Author Contributions

IC, TH, and LS contributed to conception and design of the study. LS acquired the data. LS and IC performed the statistical analysis. LS wrote the first draft of the manuscript. IC wrote sections of the manuscript and TH critically revised the manuscript. All authors contributed to manuscript revision, read and approved the submitted version.

### Conflict of Interest Statement

The authors declare that the research was conducted in the absence of any commercial or financial relationships that could be construed as a potential conflict of interest.
