# Supplementary material for: The Design Matters: How to Detect Neural Correlates of Baby Body Odors
Source: Front Neurol. 2019 Jan 16;9:1182. doi: 10.3389/fneur.2018.01182 (PMC6343458; doi:10.3389/fneur.2018.01182)
Supplement: Supplementary file 1 [file Data_Sheet_1.docx]

Supplementary Material

The design matters: How to detect neural correlates of baby body odors

Laura Schäfer*, Ilona Croy, Thomas Hummel

*** Correspondence:** Corresponding Author: laura.schaefer@uniklinikum-dresden.de

# Supplementary Figures and Tables

Supplementary Table 1

*Detailed Overview of the ROI Masks: Atlas, Working Region and Volume of each ROI*

| ROI | Atlas | Working region | Total volume for bilateral ROIs (mm) |
| --- | --- | --- | --- |
| OFC | Ibaspm 116 | frontal_sup_orb_L; frontal_sup_orb_R; frontal_mid_orb_l; frontal_mid_orb_r; frontal_inf_orb_l; frontal_inf_orb_r; frontal_mid_orb_l; frontal_mid_orb_r | 70680 |
| Insula | Ibaspm 116 | insula_L; insula_R | 29024 |
| Hippocampus | Ibaspm 116 | Hippocampus_L; Hippocampus_R | 15024 |
| Amygdala | AAL | Amygdala_L, Amygdala_R | 3744 |
| Thalamus | Ibaspm 116 | Thalamus_L; Thalamus_R | 17256 |
| ACC | Ibaspm 116 | cingulum_ant_L; cingulum_ant_R | 21704 |
| PCC | Ibaspm 116 | cingulum_post_L; cingulum_post_R | 6384 |
| Piriform cortex |  | Piriform cortex L and R built in MRIcron [McCausland Center for Brain Imaging, https://www.mccauslandcenter.sc.edu/crnl/mricron] as guided by [1] | 368 |

*Note.* All masks except for the Piriform cortex were built with WFU Pick Atlas 3.0.3 [2, 3] toolbox for SPM.

Supplementary Table 2

*Whole Brain Analysis for Short Block Design:* Main Effects of Baby Body Odor (Merged across Own and Unfamiliar Baby), Peak Height Threshold: p<.001, Cluster Extent Threshold: k>20

|  |  | Coordinates | | |  |  |  |
| --- | --- | --- | --- | --- | --- | --- | --- |
| Side | Area | X | Y | Z | Z-Value | T-Value | P_uncorr_ |
| Odor > No Odor |  |  |  |  |  |  |  |
| Right | STG | 68 | -30 | -6 | 4.19 | 5.56 | 0.000 |
| Right | Brain Stem, allocated to the trigeminal main sensory nucleus, guided by [4] | 2 | -30 | -18 | 4.01 | 5.19 | 0.000 |
| Right | OFC | 46 | 40 | -5 | 3.88 | 4.94 | 0.000 |
| Right | Anterior Insula | 36 | 16 | -7 | 3.87 | 4.92 | 0.000 |

Supplementary Table 3

*Whole Brain Analysis for Short Block Design:* Main Effect of Own Baby Body Odor, Peak Height Threshold: p<.001, Cluster Extent Threshold: k>20

|  |  | Coordinates | | |  |  |  |
| --- | --- | --- | --- | --- | --- | --- | --- |
| Side | Area | X | Y | Z | Z-Value | T-Value | P_uncorr_ |
| Own > No Odor |  |  |  |  |  |  |  |
| Right | Mediate Cingulate Cortex | 10 | -32 | 30 | 4.14 | 5.46 | 0.000 |
| Right | OFC | 44 | 44 | -10 | 3.61 | 4.46 | 0.000 |
| Left | PCC | -2 | -42 | 22 | 3.95 | 5.08 | 0.000 |
| Left | Parahippocampal Gyrus / Subiculum | -18 | -34 | -12 | 3.88 | 4.95 | 0.000 |

Supplementary Table 4

*Whole Brain Analysis for Short Block Design:* Main Effects of Unfamiliar Baby Body Odor, Peak Height Threshold: p<.001, Cluster Extent Threshold: k>20

|  |  | Coordinates | | |  |  |  |
| --- | --- | --- | --- | --- | --- | --- | --- |
| Side | Area | X | Y | Z | Z-Value | T-Value | P_uncorr_ |
| Unfamiliar > No Odor |  |  |  |  |  |  |  |
| Left | Parahippocampal Gyrus/Subiculum | -8 | -28 | -14 |  | 5.26 | 0.000 |

## Supplementary Figures

*
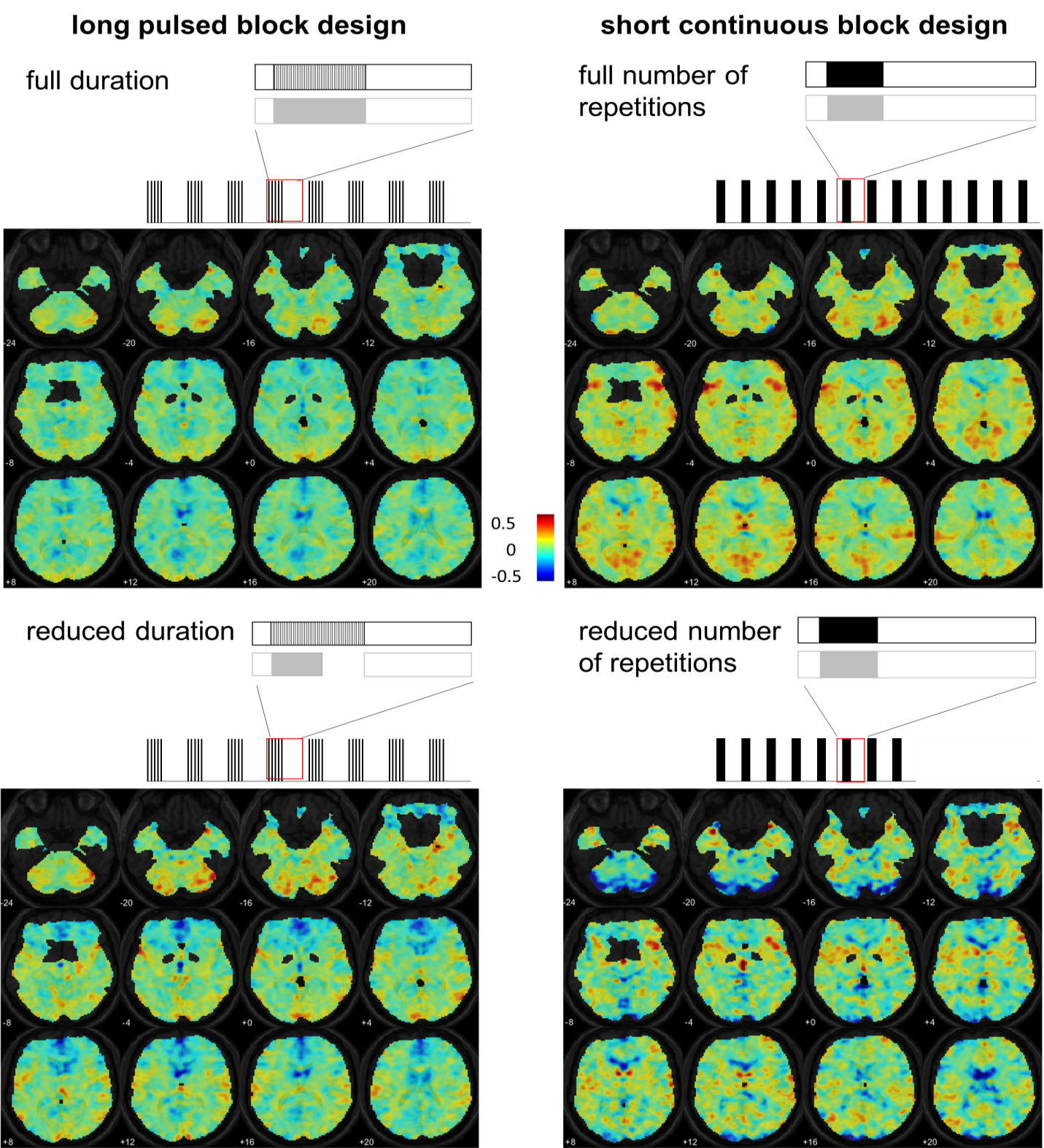
*

**Supplementary Figure 1.** Schematic visualization of design and analysis method; and true color images depicting group results of whole brain analyses. **Left panel:** long pulsed block design: 8x on-period (á 15 seconds pulsed presentation: 1 second on, 2 seconds off), 8x off-period (á 30 seconds), analyzed with full (15 seconds; upper panel, “long_full_”) and reduced (7.5 seconds; panel below, “long_reduced_”) duration of on-period. **Right panel:** short continuous block design: 13x on-period (á 6 seconds continuous presentation), 13x off-period (á 19 seconds) analyzed with full (13; panel above, “short_full_”) and reduced number of repetitions (8; panel below, “short_reduced_”). True color images of group activation (*n*=10) are presented for different design and analysis approaches representing weighted sum of related beta values. Images are displayed from z = -24 to z = +20. Color scale ranges from negative to positive intensity.

**
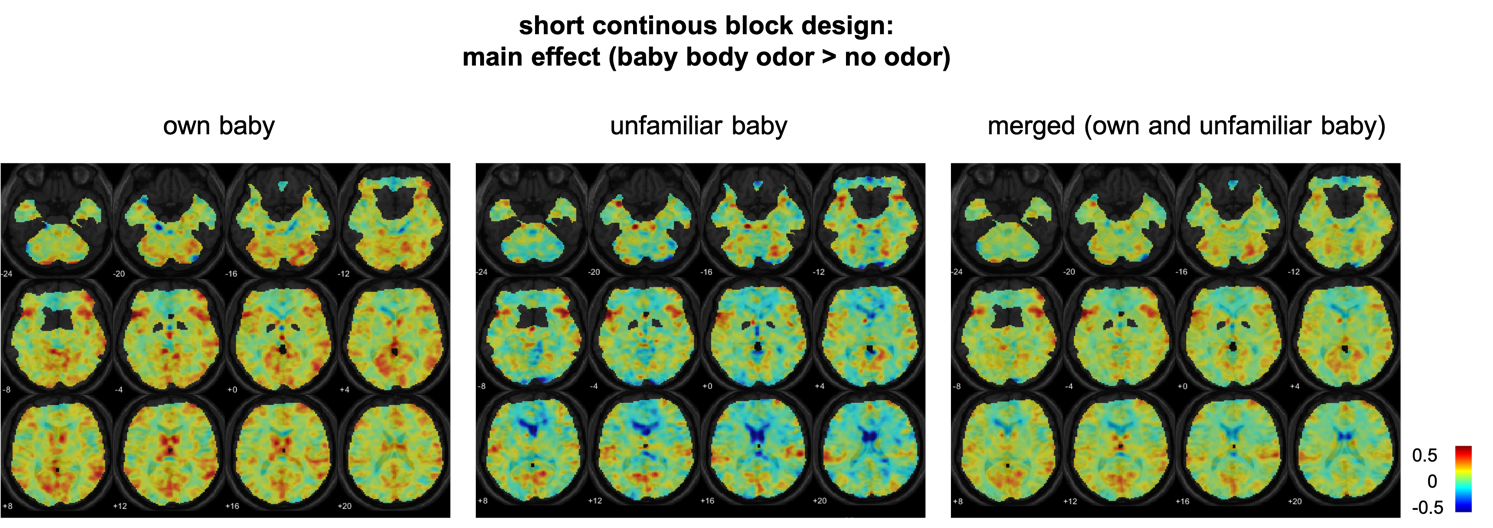
**

**Supplementary Figure 2.** True color images of group activation (*n*=10) of whole brain analyses are presented for main effects of baby body odor (own, unfamiliar, merged) for the short continuous block design representing weighted sum of related beta values. Images are displayed from z = -24 to z = +20. Color scale ranges from negative to positive intensity.

**2 Detailed description of body odor sampling and experimental presentation**

For collecting the body odor probe of their child, subjects were equipped with a study kit. The set contained an onesie (kolibri GmbH, Waiblingen, Germany, www.koli-bri.net/startseite) in the respective size of the child, a re-closable plastic zip bag, as well as odorless medical shower gel, shampoo (both EUBOS flüssig wasch+dusch, Dr. Hobein GmbH, Meckenheim, Germany, www.eubos.de) and odorless washing powder (Denkmit Vollwaschmittel Ultra Sensitive, dm-drogerie markt GmbH & Co. KG, Karlsruhe, Germany, www.dm.de). Procedure was explained in detail and subjects received a study instructions sheet for taking home. The babies had to sleep in the onesie for one night after a standardized protocol. This included precedent washing of the sheets, blankets of the child, and clothes additionally worn to the onesie with the odorless washing powder; as well as washing the child with the odorless medical shower gel the evening before the experimental night. Parents were instructed to refrain from usage of any perfumed hygiene products.

In addition, participants received an e-mail with detailed instructions for the subsequent study procedure. After the baby slept in the onesie parents were instructed to store the onesie in the zip-closed plastic bag and to bring it back to the Lab of the Psychosomatic Department of the University Hospital Dresden the morning after the experimental night. In case the delivery would take longer than 2 hours after waking up, participants were instructed to cool the onesie until it was brought to the University Hospital. At the lab, the superior part of the onesie was cut in half and frosted by -25 °C (up to eight weeks before the trial) and defrosted 1.5 hours before starting the experiment.

For the experiment, the armpit was used and stored in glass bottles connected with teflon tubes (5 m length) to the air-dilution computer controlled olfactometer [5]. The glass bottles were warmed with an fMRI compatible heating system until body temperature was reached in order to ensure an optimal distribution of the olfactory molecules. Olfactory stimulation was delivered birhinally via a silicon nose-piece (4 mm outer diameter) connected to the teflon tubing.

Parents completed additional anamnestic, demographic assessment and standardized questionnaires via an online survey (LimeSurvey, https://bildungsportal.sachsen.de/survey/) including the following questionnaires assessing bonding (Postpartal Bonding Questionnaire, PBQ, [6]), depressive symptoms (Beck Depression Inventory, BDI-II, [7]), as well as behavioral inhibition and activation (Behavioral Inhibition Scale/Behavioral Activation Scale, BIS/BAS, [8]).

**2.1 Extended sample description.**

The anamnestic and demographic assessment of the participants revealed that all children were healthy and did not suffer from any diseases, as well as there were no preterm births.

Three of 10 mothers stated complications during pregnancy (gestational diabetes*, hypertension, in-patient stay due to excessive vomiting throughout pregnancy).* Two oft ten mothers reported perinatal complications (*kidney congestion*, *abdominal delivery, sudden decrease of heartbeat*)*.* One mother stated postnatal complications (*jaundice*)*.*

**3 Supplementary References**

1. Zelano, C., et al., *Attentional modulation in human primary olfactory cortex.* Nature neuroscience, 2005. **8**(1): p. 114.

2. Maldjian, J.A., P.J. Laurienti, and J.H. Burdette, *Precentral gyrus discrepancy in electronic versions of the Talairach atlas.* Neuroimage, 2004. **21**(1): p. 450-455.

3. Maldjian, J.A., et al., *An automated method for neuroanatomic and cytoarchitectonic atlas-based interrogation of fMRI data sets.* Neuroimage, 2003. **19**(3): p. 1233-1239.

4. Elliott, L., et al., *Gray’s Clinical Neuroanatomy: The Anatomic Basis for Clinical Neuroscience*. 2011, Philadelphia: Elsevier Saunders.

5. Sommer, J.U., et al., *A mobile olfactometer for fMRI-studies.* Journal of neuroscience methods, 2012. **209**(1): p. 189-194.

6. Brockington, I., C. Fraser, and D. Wilson, *The postpartum bonding questionnaire: a validation.* Archives of women's mental health, 2006. **9**(5): p. 233-242.

7. Hautzinger, M., F. Keller, and C. Kühner, *Beck depressions-inventar (BDI-II)*. 2006: Harcourt Test Services Frankfurt.

8. Strobel, A., et al., *Eine deutschsprachige Version des BIS/BAS-Fragebogens von Carver und White.* Zeitschrift für Differentielle und diagnostische Psychologie, 2001.
